# Local nebulization of 1α,25(OH)_2_D_3_ attenuates LPS-induced acute lung inflammation

**DOI:** 10.1186/s12931-022-01997-9

**Published:** 2022-03-29

**Authors:** Jef Serré, Carolien Mathyssen, Tom Tanjeko Ajime, Tobias Heigl, Lieve Verlinden, Karen Maes, Annemieke Verstuyf, Didier Cataldo, Jeroen Vanoirbeek, Bart Vanaudenaerde, Wim Janssens, Ghislaine Gayan-Ramirez

**Affiliations:** 1 Laboratory of Respiratory Diseases and Thoracic Surgery (BREATHE), Department of Chronic Diseases and Metabolism (CHROMETA), KU Leuven, Herestraat 49, O&NI bis, Box 706, B-3000 Leuven, Belgium; 2 Clinical and Experimental Endocrinology, Department of Chronic Diseases and Metabolism (CHROMETA), KU Leuven, Leuven, Belgium; 3 Laboratory of Tumor and Development Biology, GIGA-Cancer, University of Liège, Liège, Belgium; 4 Department of Respiratory Diseases, CHU of Liège, University of Liège, Liège, Belgium; 5 Centre for Environment and Health, Department of Public Health and Primary Care, KU Leuven, Leuven, Belgium; 6 Clinical Department of Respiratory Diseases, UZ Leuven, Leuven, Belgium

**Keywords:** Vitamin D deficiency, Acute lung inflammation, Local vitamin D nebulization

## Abstract

**Background:**

Evidence supports a critical role of vitamin D status on exacerbation in chronic obstructive pulmonary disease, indicating the need to avoid vitamin D deficiency in these patients. However, oral vitamin D supplementation is limited by the potential risk for hypercalcemia. In this study, we investigated if local delivery of vitamin D to the lungs improves vitamin D-mediated anti-inflammatory action in response to acute inflammation without inducing hypercalcemia.

**Methods:**

We studied vitamin D sufficient (VDS) or deficient (VDD) mice in whom 1α,25(OH)_2_D_3_ (0.2 μg/kg) or a vehicle followed by lipopolysaccharide (LPS 25 µg) were delivered to the lung as a micro-spray.

**Results:**

Local 1α,25(OH)_2_D_3_ reduced LPS-induced inflammatory cells in bronchoalveolar lavage (BAL) in VDS (absolute number of cells: − 57% and neutrophils − 51% p < 0.01) and tended to diminish LPS-increased CXCL5 BAL levels in VDS (− 40%, p = 0.05) while it had no effect on CXCL1 and CXCL2 in BAL and mRNA in lung of VDS and VDD. It also significantly attenuated the increased IL-13 in BAL and lung, especially in VDD mice (− 41 and − 75%, respectively). mRNA expression of Claudin-18 in lung was significantly lower in VDS mice with local 1α,25(OH)_2_D_3_ while Claudin-3, -5 and -8 mRNA levels remained unchanged. Finally, in VDD mice only, LPS reduced lung mRNA expression of adhesion junction Zona-occludens-1, in addition to increasing uric acid and total protein in BAL, which both were prevented by local 1α,25(OH)_2_D_3_.

**Conclusion:**

Under normal levels of vitamin D, local 1α,25(OH)_2_D_3_ nebulization into the lung efficiently reduced LPS induction of inflammatory cells in BAL and slightly attenuated LPS-increase in CXCL5. In case of severe vitamin D deficiency, although local 1α,25(OH)_2_D_3_ nebulization failed to significantly minimize cellular inflammation in BAL at this dose, it prevented epithelial barrier leakage and damage in lung. Additional research is needed to determine the potential long-term beneficial effects of local 1α,25(OH)2D3 nebulization on lung inflammation.

**Supplementary Information:**

The online version contains supplementary material available at 10.1186/s12931-022-01997-9.

## Introduction

Chronic obstructive pulmonary disease (COPD) is characterized by a progressive airflow limitation driven by an abnormal chronic lung inflammatory response to noxious particles and gases. Cigarette smoking is the major risk factor in the development of COPD in industrialized countries, but factors such as genetics, age, gender, lung development and malnutrition are also involved [1]. As COPD severity progresses, patients suffer from increased incidence of acute exacerbations (AECOPD) defined as an acute inflammatory flare-up of the disease that temporarily worsens respiratory symptoms (e.g. coughing and dyspnea) and lung function requiring a change in medication [1]. In more severe cases, this results in emergency hospitalization which is associated with a 25% increased one-year mortality. A permanent loss in forced expiratory volume in one second (FEV_1_) was also found to occur in 25% of patients after AECOPD [2]. Some patients are more prone to AECOPD with frequent exacerbators (> 2 exacerbations/year), who are found in all severities of the disease [3], becoming more housebound and showing annually a 25% steeper FEV_1_ decline than infrequent exacerbators [4, 5]. Clearly, AECOPD represent important events in the course of the disease with serious repercussions on health status, lung function and quality of life.

AECOPD are currently managed with inhaled bronchodilators (e.g., long acting beta_2_ agonist (LABA) and long acting antimuscarinic antagonist (LAMA)), corticosteroids and antibiotics. Patients with frequent exacerbation may also be prescribed maintenance therapy, such as inhaled corticosteroids, and/or a course of macrolides, such as azithromycin. However, not all patients with AECOPD benefit from such treatment so there remains a need to improve therapeutics to prevent or reduce AECOPD.

Recent studies have underscored the importance of vitamin D status for lung health. Interestingly, vitamin D deficiency (VDD) defined as 25(OH)D serum levels < 20 ng/ml, is highly prevalent in COPD patients compared to healthy smokers and becomes even more common as the COPD severity increases and lung function worsens [6, 7]. Clinical studies have also reported an inverse relationship between 25(OH)D serum levels and frequent upper or lower respiratory tract infections [8, 9], community-acquired pneumonia [10], tuberculosis [11], and COVID19 [12]. In COPD, observational studies indicated that severe vitamin D deficiency (25(OH)D serum levels < 10 ng/ml) correlated with frequent exacerbations [13, 14]. A recent meta-analysis showed that oral vitamin D supplementation in COPD patients did reduce the annual exacerbation frequency, but only in severe vitamin D deficient COPD patients (< 10 ng/ml 25(OH)D serum levels) [15]. Taking together, these data support an essential role for vitamin D status on exacerbations in COPD, indicating the need to minimize the effects of VDD in these patients. However, the major problem with vitamin D supplementation is the potential risk of hypercalcemia, which limits the use of higher or frequent oral doses of vitamin D. Alternatively, vitamin D analogues with less calcemic effects could be considered, but it remains difficult to determine dosage to achieve sufficient levels of active vitamin D within the lung. However, caution should also be taken as some of these clinically approved analogues might still induce adverse effects including hypercalcemia in treated patients [16].

An appealing approach would be the local delivery of 1α,25(OH)_2_D directly to the lungs knowing that airway epithelial cells express the vitamin D receptor (VDR) and the vitamin D enzymes CYP27B1 necessary to convert 25(OH)D to 1α,25(OH)_2_D as well as CYP24A1, which catabolizes 25(OH)D and 1α,25(OH)_2_D [17, 18]. This is particularly interesting as in the lungs vitamin D can regulate genes associated with oxidative stress [19], inflammation [20, 21], T-cell development [20, 22], antimicrobial peptide production [23], lung development [24, 25] and remodeling [26, 27]. These in vitro data support the concept that local delivery of 1α,25(OH)_2_D_3_ directly to the lungs might be an efficient way to counter the effects of inflammation. We, therefore, hypothesized that local administration of 1α,25(OH)_2_D_3_ directly to the lungs would improve vitamin D-mediated anti-inflammatory action in response to acute inflammation without inducing hypercalcemia and we examined whether such an approach was applicable in a vitamin D deficient setting. This was explored using vitamin D sufficient or deficient mice in whom 1α,25(OH)_2_D_3_ followed by lipopolysaccharide were delivered to the lung as a micro spray.

## Materials and methods

### Study design

**Fig. 1:**
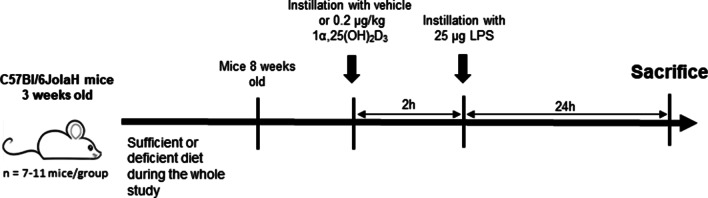
Study design. Three-week old male mice were fed a vitamin D deficient or standard control diet during the whole study period. At the age of 8–9 weeks, mice were instilled via an aerosolizer syringe with 0.2 μg/kg 1α,25(OH)_2_D_3_ or a vehicle 2 h prior to a 25 μg LPS instillation and measurements were performed 24 h later

Three-week old male C57Bl/6JolaH mice were housed in individually ventilated cages and kept in an ultraviolet light-free environment with a 12/12 h light–dark cycle. Mice were fed a vitamin D deficient diet containing 20% w/w lactose, 2% w/w calcium, and 1.25% w/w phosphorus to maintain normal calcium and phosphorus serum levels (VDD, n = 11 mice/group) (TD.87095, Envigo Teklad custom diet, Madison, Wis. USA) or standard control diet (VDS, n = 7 mice/ group) (Ssniff Bioservices, Uden, The Netherlands) throughout the study. At 8–9 weeks of age, mice were mildly anesthetized with isoflurane and administered with either 0.2 μg/kg 1α,25(OH)_2_D_3_ or the vehicle with a MicroSprayer® Aerosolizer (Penn-Century™, Wyndmoor, USA) 2 h prior to a 25 μg LPS (1 mg/kg, O111:B4 E. coli, 054M4066V) instillation (Fig. 1). The doses of LPS and 1α,25(OH)_2_D_3_ were first established in pilot studies examining the dose response effects of LPS alone and then combined with 1α,25(OH)_2_D_3_ on total cell count in broncho-alveolar lavage. The dose of LPS was chosen to induce a similar level of inflammation in the VDS and VDD mice. Experiments were approved by the Ethical Committee of Animal Experiments of the KU-Leuven (P082/2016).

### Broncho-alveolar lavage

At sacrifice, mice were anesthetized intraperitoneally with a mixture of Xylazine (10 mg/kg, Rompun®, Bayer, Belgium) and Ketamine (100 mg/kg, Ketalar®, Pfizer, Belgium), tracheotomized and bronchoalveolar lavage (BAL) was performed using first 500 μl and then 3 times 1000 μl of saline (B. Braun Medical, Diegem, Belgium). The supernatant of the first fraction was collected for cytokine analysis via centrifugation (10 min, 1000*g*, 4 °C), while total cell count was determined on all pooled cell fractions using a Bürker hemocytometer with trypan blue. Differential cell counts were determined after centrifugation (6 min, 300*g*) of BAL cells onto microslides with cytospins (Shandon, TechGen, Zellik, Belgium) and staining with Diff-Quick® (Medical Diagnostics, Düdingen, Germany). On each slide, 100 cells were counted 3 times to quantify the various cell populations (VDS n = 7/group, VDD n = 11/group).

### Lung histology

The left lung was fixed at a constant hydrostatic pressure of 25 cm H_2_O in 6% paraformaldehyde for 24 h (VDS n = 7/group, VDD n = 11/group). The lung was subsequently dehydrated and embedded in paraffin. Sagittal sections were stained with Hematoxylin & Eosin to examine lung inflammation.

### 25(OH)D and calcium serum levels

Venous blood was collected from the vena cava and kept at room temperature for 1 h, centrifuged at 1500*g* for 15 min (4 °C) and serum was collected and stored at − 80 °C. Serum 25(OH)D levels were measured by liquid chromatography tandem-mass spectrometry (LC, Shimadzu, and MS, Qtrap 5500, Sciex) at the clinical laboratory, UZ Leuven. Samples (n = 5/group) were extracted with methanol mixture spiked with internal standard 25-OH Vit-D3-d6. Serum calcium levels (VDS n = 7/group, VDD n = 11 and 9 for vehicle and 1α,25(OH)_2_D_3_, respectively) were analyzed by Olympus AU640 Chemistry Analyzers (Beckman Coulter).

### Pro-inflammatory mediators

A lung tissue sample was weighed and homogenized in 1 × PBS. Pro-inflammatory mediators such as IFN-γ, IL-1β, IL-4, IL-6, IL-10, IL-13, IL-17A, TNF-α, CXCL1 and CXCL2 were analyzed in cell-free BAL (VDS: n = 7/group, VDD: n = 11 and 9 for vehicle and 1α,25(OH)_2_D_3_, respectively), lung homogenate (n = 6/group) and serum (n = 6/group) with a MSD U-plex® multiplex assay (Meso Scale Discovery®, Rockville, USA). CXCL5 was measured in cell-free BAL via ELISA kit (VDS: n = 7/group, VDD n = 11 and 9 for vehicle and 1α,25(OH)2D3, respectively) (DY443, R&D systems, Abingdon, UK). Assay detection range is provided in Additional file 4: Table S2 for each mediator. The rationale to assess IL-13 in this model was based on previous studies showing that IL-13 is playing a protective role in lung injury and sepsis [28–30], it can interact with vitamin D pathway while increasing the vitamin D-mediated expression of the antimicrobial cathelicidin expression in bronchial epithelial cells [31] and its expression in response to LPS is inhibited by vitamin D [32].

### mRNA expression levels of inflammatory mediators and tight junctions

mRNA expression levels were measured with qRT-PCR as previously described [33]. Briefly, the right lungs were snap frozen in liquid nitrogen and stored at -80 °C. Lungs were homogenized in TRIzol (ThermoFisher scientific) and total RNA was extracted via the RNeasy mini kit (Qiagen, Venlo, the Netherlands). One µg of total RNA was reverse transcribed to cDNA, with Superscript III reverse transcriptase (Invitrogen) while using random hexamer primers. The quantitative PCR amplification was performed using Platinum SYBR Green qPCR SuperMix-UDG with the Eco Real-time PCR thermocycler (Illumina, Eindhoven, the Netherlands) (VDS: n = 6 and 7, VDD: n = 10 and 9 for vehicle and 1α,25(OH)_2_D_3_, respectively). Ribosomal protein L27 (RPL27) was used to normalize the data. All primers in Additional file 3: Table S1 were tested within the efficiency range of 90–105%.

### Lung permeability and epithelial damage

Lung permeability was assessed via measurement of total protein concentration in cell-free BAL using the Bradford method (Bio-Rad, Temse, Belgium) (VDS: n = 6 and 5, VDD: n = 10 and 9 for vehicle and 1α,25(OH)_2_D_3_, respectively) and surfactant protein (SP)-D (DY6839-05, R&D Systems) in the serum (VDS: n = 6 and 7, VDD: n = 8 and 9 for vehicle and 1α,25(OH)_2_D_3_, respectively), according to manufacture protocol. Epithelial damage was assessed via measurement of uric acid in cell-free BAL (VDS: n = 7/group, VDD: n = 10 and 9 for vehicle and 1α,25(OH)2D3, respectively) (Amplex™ red uric acid/uricase assay kit, Invitrogen).

### Statistical analysis

Datasets were analyzed using GraphPad Prism 8.1.1 for windows (GraphPad Software, San Diego, USA). A Kruskal–Wallis test followed by Dunn’s post hoc test was used to compare the data between the 4 groups while a Mann Whitney test was used for comparison between 2 groups. Differences were considered significant when p-values were less than 0.05. Data are presented as median ± IQR.

## Results

### Serum 25(OH)D and Calcium levels

25(OH)D serum levels in vitamin D deficient (VDD) mice were seven to nine-fold lower (< 2 ng/ml) than in vitamin D sufficient (VDS) mice (vehicle: 17.4 (12.2–18.7) ng/ml and 1α,25(OH)_2_D_3_: 14.4 (13.4–17.4) ng/ml, n = 5/groups). Serum calcium concentrations were within normal range in VDS (vehicle: 8.9 (8–9.2) mg/dl and 1α,25(OH)_2_D_3_: 8.7 (8.3–9.2) mg/dl, n = 7/group) and VDD mice (vehicle: 9.7 (9.5–10.3) mg/dl, n = 11 and 1α,25(OH)_2_D_3_: 9.9 (8.8–10.5) mg/dl, n = 9) with no statistical differences between the groups.

### Airway inflammation in BAL

**Fig. 2:**
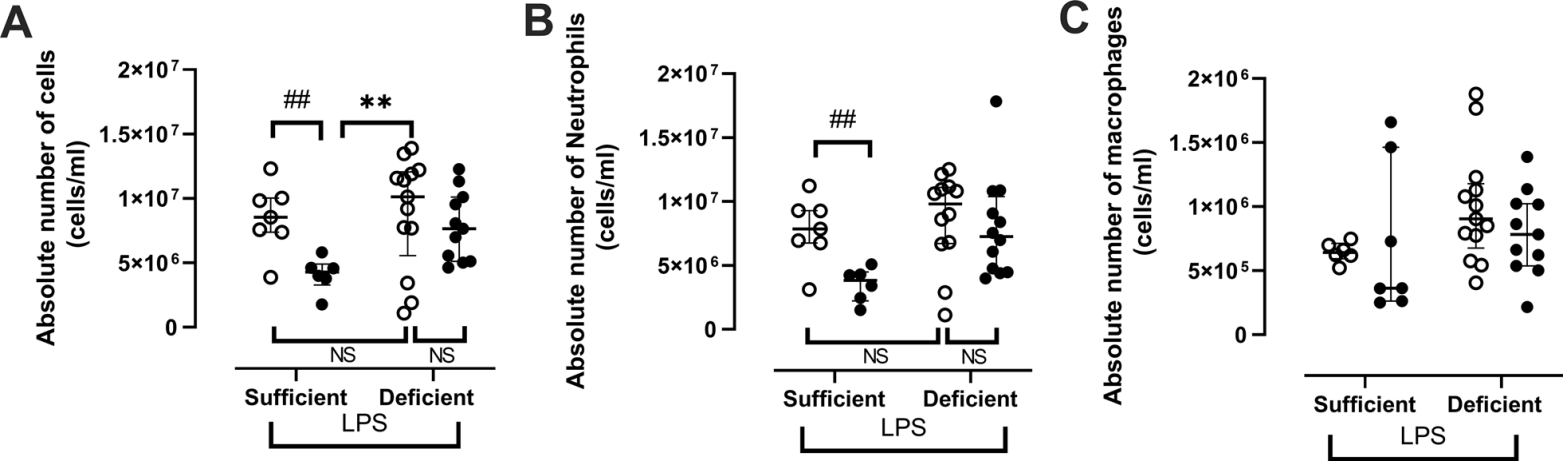
Absolute number of total cells (**A**), neutrophils (**B**) and macrophages (**C**) in BAL of vitamin D sufficient or deficient mice treated either with vehicle (open circles) or 1α,25(OH)_2_D_3_ (closed circles) followed by LPS. NS: not significant, **: p < 0.01 (Kruskal–Wallis); ##: p < 0.01 (Mann–Whitney). Data are expressed as median ± IQR

Absolute number of cells in BAL increased by 26% (NS) in vehicle-nebulized VDD compared to vehicle-nebulized VDS (Fig. 2A). Similarly, a 25% increase in absolute number of neutrophils (NS) were observed in vehicle-nebulized VDD compared to vehicle-nebulized VDS (Fig. 2B) while no differences were found between groups in absolute number of macrophages (Fig. 2C). Local intratracheal nebulization of 1α,25(OH)_2_D_3_ significantly reduced LPS-induced inflammation by 57% (p < 0.01) for absolute number of cells (Fig. 2A) and by 51% (p < 0.01) for neutrophils (Fig. 2B) in VDS compared to vehicle-treated VDS. In VDD, 1α,25(OH)_2_D_3_ nebulization reduced absolute number of cells by 29% (Fig. 2A) and neutrophils by 26% (Fig. 2B) compared to vehicle-nebulized VDD but these reductions failed to reach statistical significance. Absolute number of cells was significantly lower in 1α,25(OH)_2_D_3_ nebulized-VDS mice compared to vehicle-treated VDD animals (p < 0.01, Kruskal–Wallis) (Fig. 2A).

### Lung inflammation

Sign of inflammation with accumulation of numerous inflammatory cells notably in the alveolar cavity was observed in lung on histological section stained with H&E. There was also clear evidence for neutrophilic inflammation. Representative examples of H&E stained lung section are depicted in Additional file 1: Figure S1, Additional file 2 for the 4 groups.

### Chemoattractant in lungs

**Fig. 3:**
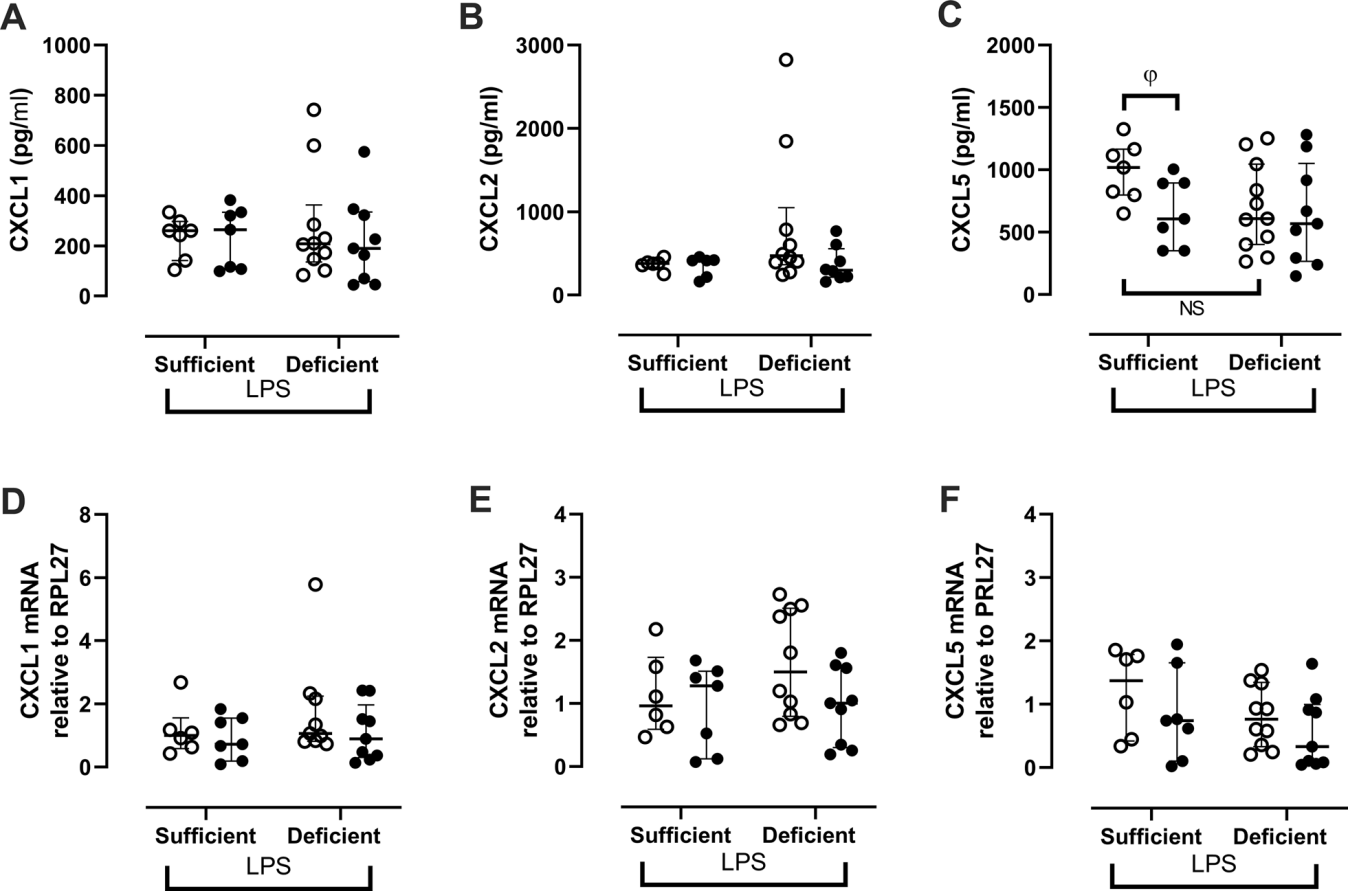
Chemokines **A** CXCL1, **B** CXCL2 and **C** CXCL5 in BAL and mRNA expression of **D** CXCL1, **E** CXCL2 and **F** CXCL5 in lung homogenate in vitamin D sufficient or deficient mice treated either with vehicle (open circles) or 1α,25(OH)_2_D_3_ (closed circles) followed by LPS. mRNA data expressed relative to RPL27. NS: not significant, φ: p = 0.05 (Mann Whitney). Data are expressed as median ± IQR

In BAL, CXCL1 and CXCL2 concentrations were similar in vehicle-nebulized VDD and VDS (Fig. 3A, B). Although CXCL5 concentration was slightly decreased in vehicle-nebulized VDD compared to vehicle-nebulized VDS, this reduction failed to reach statistical significance (Fig. 3C). Local nebulization with 1α,25(OH)_2_D_3_ tended to reduce CXCL5 levels in VDS compared to vehicle-nebulized VDS (− 40%, p = 0.05; Fig. 3C), but had no effects on other chemokines in both VDS and VDD. In lungs, mRNA expression of CXCL1, CXCL2 and CXCL5 were similar between the different groups (Fig. 3D, E, F).

### Inflammatory cytokines in lungs

Concentrations of IL-6, IL-10, IL-17A, TNF-α and IFN-ϒ were similar between groups in both cell free BAL fluid and in lung homogenate (Table 1). IL-1β levels in BAL fluid were significantly higher in vehicle-nebulized VDD compared to vehicle-nebulized VDS and local nebulization with 1α,25(OH)_2_D_3_ did not affect these concentrations (Table 1). Finally, IL-13 was significantly increased in BAL and lung of vehicle-nebulized VDD compared to vehicle-nebulized VDS (p < 0.05) (Table 1). Local nebulization with 1α,25(OH)_2_D_3_ significantly reduced IL-13 in BAL (-41%, p < 0.01) and lung of VDD (-75%, p < 0.05) compared to vehicle-nebulized VDD (Table 1).

**Table 1:** Pro-inflammatory cytokines measured in BAL fluid and lung homogenate of vitamin D sufficient or deficient mice treated either with vehicle or 1α,25(OH)_2_D_3_ followed by LPS. Interleukin (IL), Tumor necrosis factor-α (TNF-α) and interferon-γ (IFN-γ) expressed as pg/ml for BAL fluid and pg/µg for lung homogenate

	BAL				LUNG			
	Sufficient		Deficient		Sufficient		Deficient	
	Vehicle	1α,25(OH)_2_D_3_	Vehicle	1α,25(OH)_2_D_3_	Vehicle	1α,25(OH)_2_D_3_	Vehicle	1α,25(OH)_2_D_3_
IL-1β	29.7 (22.2–33.1)	25.7 (16.4–29.4)	42.9 (31.5–52.1) +	30.4 (11.7–46.7)	8802 (3366–19,353)	5595 (2883–15,263)	4590 (3514–16,624)	4799 (659–11,665)
IL-6	5603 (3747–7703)	3983 (2241–4562)	3795 (1787–8395)	3027 (537–7070)	1878 (755–2811)	1397 (600–6790)	5731 (851–18,101)	1125 (307–2454)
IL-10	9.8 (6.4–13.3)	7.5 (6.6–13.7)	18.7 (11.8–68.0)	14.5 (9.7–33.5)	30.6 (14.5–49.8)	39.5 (15.9–47.5)	71.4 (17.8–179)	14.9 (5.6–38.1)
IL-13	15.4 (13.7–19.3)	13.9 (11.3–17.7)	20.9 (16.4–30.5)*	14.8 (12.2–16.0)^##^	88.2 (36.6–112)	80.2 (29.8–131.7)	135 (93.4–201.4) +	34.1 (14.4–53.7)^#^
IL-17A	0.8 (0.6–1.2)	1.2 (0.5–2.8)	1.4 (0.5–3.2)	0.6 (0.3–1.2)	3.9 (2.7–5.6)	3.7 (0.9–19.7)	5.9 (2.6–24)	2.4 (1.2–5.9)
TNF-⍺	987 (682–1223)	811 (405–1152)	1036 (505–1459)	1115 (550–1373)	915 (434–1881)	736 (302–1529)	946 (550–2161)	591 (148–1051)
IFN-γ	2.34 (0.76–3.97)	2.98 (0.37–16.53)	1.16 (0.57–8.28)	1.03 (0.34–18.47)	3.9 (1.6–6.3)	3.2 (1.2–24.7)	3.8 (2.6–9.1)	2.5 (0.7–14.3)

*: p < 0.05 vehicle deficient vs vehicle sufficient (Kruskal–Wallis); #: p < 0.05 1α,25(OH)_2_D_3_ deficient vs vehicle deficient (Kruskal–Wallis); + : p < 0.05 vehicle deficient vs vehicle sufficient (Mann Whitney); ##: p < 0.01 1α,25(OH)_2_D_3_ deficient vs vehicle deficient (Mann Whitney). Data are expressed as Median ± IQR, with n = 7/group (VDS) n = 11 (vehicle-VDD) and n = 9 (1α,25(OH)_2_D_3_-VDD) for measurements in BAL and n = 6/group for measurements in lung

**Table 2:** Cytokine serum levels in vitamin D sufficient or deficient mice treated either with vehicle or 1α,25(OH)_2_D_3_ followed by LPS. Interleukin (IL), Tumor necrosis factor-α (TNF-α) and interferon-γ (IFN-γ) expressed as pg/ml, - : below detection limit

	Serum				
	Sufficient		Deficient		
	Vehicle	1α,25(OH)_2_D_3_	Vehicle		1α,25(OH)_2_D_3_
IL-1β	–	–	–		–
IL-6	115 (78.5–161)	189 (149–433)^#^	152 (77.5–367)		125 (89–195)
IL-10	25.3 (16.8–42.8)	78.5 (43.3–157)^#^	36.9 (22.4–816)		25.2 (19.0–34.6)
IL-13	–	–	–		–
IL-17A	0.3 (0.2–0.4)	0.5 (0.3–0.7)	1.4 (0.06–10.3)		0.2 (0.1–0.5)
TNF-⍺	19.0 (12.4–27.0)	21.8 (16.7–36.2)	52.7 (23.7–143)		17.3 (15.5–22.6)
IFN-γ	0.9 (0.5–1.2)	1.5 (0.5–19.0)	3.8 (0.5–20.8)		3.8 (0.4–8.3)

#: p < 0.05 1α,25(OH)_2_D_3_ sufficient vs vehicle sufficient (Mann Whitney). Data are expressed as median ± IQR with n = 6/group

### Tight and adhesion junctions in lung

**Fig. 4:**
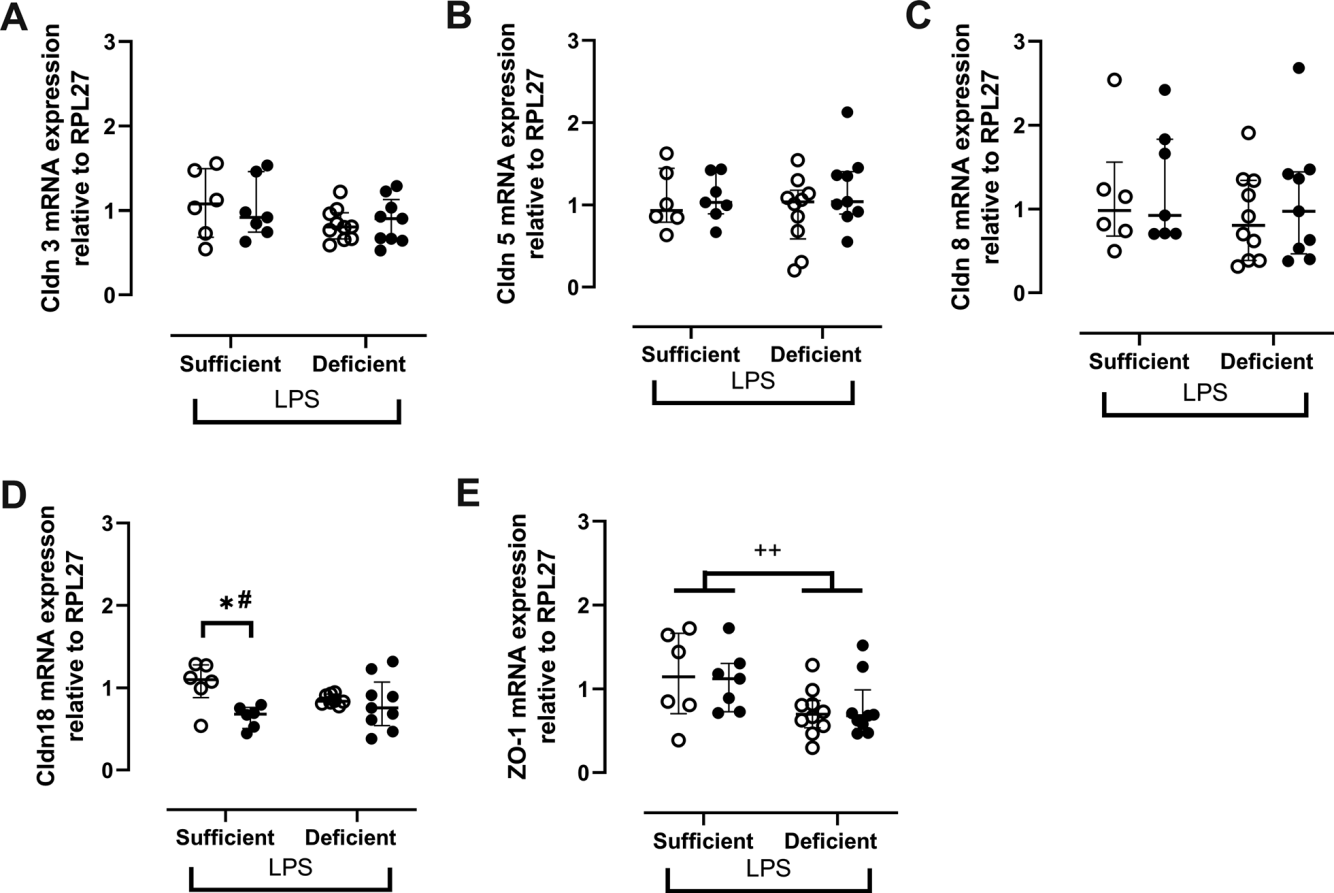
Lung mRNA expression of tight junctions namely claudin (Cldn) 3 (**A**), 5 (**B**), 8 (**C**) and 18 (**D**) and adhesion junction Zona occludens-1 (ZO-1) (**E**) in vitamin D sufficient or deficient mice treated either with vehicle (open circles) or 1α,25(OH)_2_D_3_ (closed circles) followed by LPS. mRNA data expressed relative to RPL27. * and #: p < 0.05 (Kruskal–Wallis and Mann Whitney); +  + : p < 0.01 (Mann Whitney). Data are expressed as median ± IQR

The mRNA expression of tight junctions, Claudin 5, 8 and 18 were similar between VDS and VDD (Fig. 4A-D) while mRNA expression of zona occludens-1 (ZO-1) was significantly reduced in VDD even when pre-treated with 1α,25(OH)_2_D_3_ compared to VDS (p < 0.01) (Fig. 4E). Claudin 18 mRNA expression was downregulated in VDS when pre-treated with 1α,25(OH)_2_D_3_ compared to vehicle-treated VDS (-38%, p < 0.05; Fig. 4D) while no changes in claudin 18 mRNA were seen in VDD (Fig. 4D).

### Lung permeability and epithelial damage

**Fig. 5:**
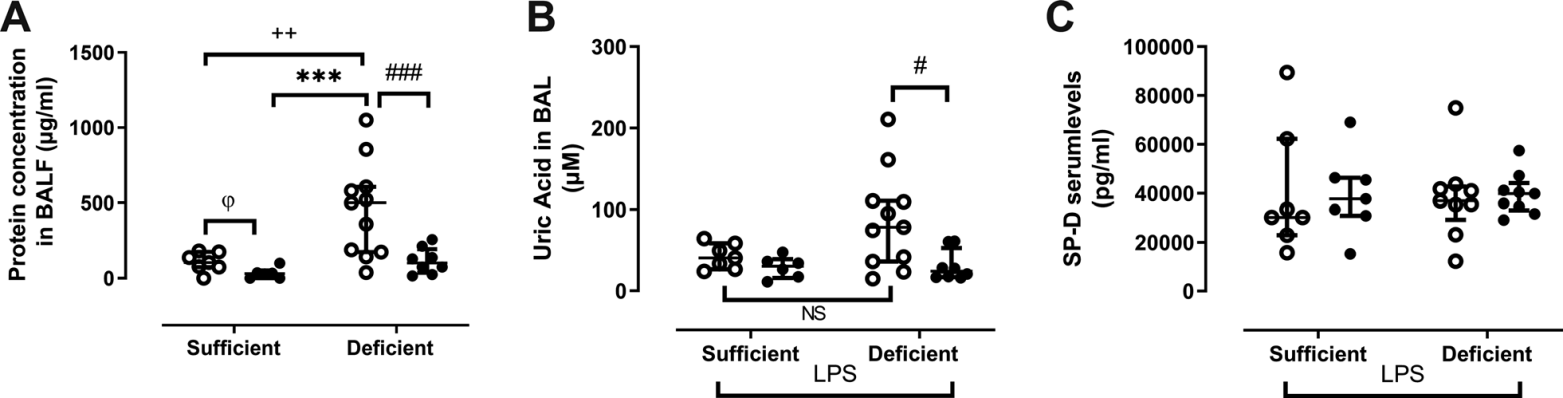
Protein (**A**) and uric acid (**B**) concentration in BAL fluid expressed as µg/ml and µM, respectively and serum Surfactant protein-D (SP-D) concentration expressed as pg/ml measured in vitamin D sufficient or deficient mice treated either with vehicle (open circles) or 1α,25(OH)_2_D_3_ (closed circles) followed by LPS. NS: not significant, ***: p < 0.001 (Kruskal–Wallis); φ: p = 0.05 (Mann Whitney); #: p < 0.05 (Mann Whitney); ###: p < 0.001 (Mann Whitney) +++ : p < 0.005 (Mann Whitney). Data are expressed as median ± IQR

Protein content in cell-free BAL fluid of vehicle-nebulized VDD increased five-fold compared to vehicle-nebulized VDS (p < 0.01) and 16-fold compared to 1α,25(OH)_2_D_3_ nebulized-VDS (p < 0.001) (Fig. 5A). Local nebulization with 1α,25(OH)_2_D_3_ significantly reduced protein content in VDD compared to vehicle-nebulized VDD (-80%, p < 0.001, Fig. 5A) and tended to reduce it in VDS compared to vehicle-nebulized VDS (p = 0.05, Fig. 5A). Uric acid in BAL increased two-fold in vehicle-treated VDD compared to vehicle-treated VDS (NS) and showed 60% reduction with local 1α,25(OH)_2_D_3_ administration (p < 0.05, Fig. 5B). No significant differences in SP-D serum levels were found between the groups (Fig. 5C).

### Systemic cytokine inflammation

Serum levels of IL-17A, TNF-α and IFN-ϒ were similar between groups, while IL-1β and IL-13 fell below the detection limit (Table 2). IL-6 and IL-10 serum levels were significantly increased in VDS with local 1α,25(OH)_2_D_3_ nebulization compared to vehicle-nebulized VDS (p < 0.05) while no changes were observed in VDD (Table 2).

## Discussion

This is the first study examining local delivery of 1α,25(OH)_2_D_3_ directly to the lungs via nebulization in VDS or VDD mice as a potential strategy to minimize acute lung inflammation induced by LPS. In agreement with our hypothesis, local 1α,25(OH)_2_D_3_ nebulization into the lung attenuated LPS-induced inflammation without inducing serum hypercalcemia. Anti-inflammatory action of locally delivered 1α,25(OH)_2_D_3_ was observed on inflammatory cell count in BAL and solely on pro-inflammatory cytokine IL-13 in BAL and lung. In addition, local nebulization with 1α,25(OH)_2_D_3_ was able to slightly reduce inflammation in VDD, and to significantly protect from exaggerated epithelial barrier damage. These data suggest that local nebulization of 1α,25(OH)_2_D_3_ into the lung might represent an interesting strategy to avoid acute lung inflammation in VDS but also to reduce epithelial damage associated with VDD.

A recent systematic review with meta-analysis demonstrated an overall safe use of oral vitamin D3 supplementation in all participants which restored 25(OH)D serum levels to sufficiency (> 30 ng/ml). Interestingly, it also demonstrated an overall protective effect against acute respiratory tract infections (ARTI) [34]. Severe vitamin D deficient individuals at baseline gained the most protection against ARTI when receiving daily or weekly vitamin D3 instead of a monthly high bolus [34]. Similarly, a meta-analysis in vitamin D3 supplemented COPD-patients also reported a protective effect against exacerbation rate with a 45% reduction, but only in patients with severe vitamin D deficiency (< 10 ng/ml 25(OH)D serum levels) at baseline [15]. For oral vitamin D administration to be more efficient, higher doses might be needed. However, increasing oral vitamin D3 dosage and frequency is not recommended because of its detrimental effect on calcium absorption in the intestines [35], potentially increasing serum calcium levels to toxic concentrations (> 10 mg/dl). Furthermore, the potential benefit of oral vitamin D3 supplementation is limited in acute situations, as it only adjusts 25(OH)D homeostasis over the long-term and, as such, cannot directly participate in acute setting without first a conversion to 25(OH)D and 1α,25(OH)_2_D. Alternatively, the use of vitamin D analogues with similar activity as vitamin D but with less calcemic effects can be considered. While several vitamin D analogues have been approved for use in clinic for the treatment of psoriasis, secondary hyperparathyroidism or osteoporosis [36], to the best of our knowledge, no vitamin D analogue is currently used in the clinic for the treatment of lung disease. However, adverse effects after treatment with vitamin D analogues have been reported with hypercalcemia being the most common in at least one-third of the patients taking systemic calcitriol [16]. As such, better routes of administration of vitamin D need to be considered which reduce these detrimental effects while maintaining efficacy.

In this study, we explored the potential of 1α,25(OH)_2_D_3_ delivered locally into the lungs to improve vitamin D-mediated anti-inflammatory action during an acute respiratory inflammation induced by LPS. The idea behind this approach is based on in vitro studies indicating that many cells in the lungs express VDR and respond to exogenous vitamin D [18, 23]. In addition, some of these cells also express the CYP27B1 enzyme to convert 25(OH)D to 1α,25(OH)_2_D and CYP24A1 to catabolize both 25(OH)D and 1α,25(OH)_2_D. In addition, some cells can even constitutively produce 1α,25(OH)_2_D or when stimulated by inflammatory triggers [17, 18, 37]. In particular, airway epithelial cells express VDR but also high levels of activating CYP27B1 and low levels of CYP24A1 at baseline and they constitutively generate 1α,25(OH)_2_D [17, 18, 38]. Importantly, we recently reported that VDR localization was restricted to the apical layer of the bronchial epithelial cells while VDR was completely absent in the basal cells of the epithelium and also in the vascular endothelial cells on histology of COPD and donor human lungs [18]. This observation further supports the idea that local delivery of 1α,25(OH)_2_D to the airway epithelium might be a promising strategy to target lung inflammation. This is further reinforced by our previous finding showing that pulmonary vascular endothelial cells express high levels of CYP24A1 [18]. As such, it might be expected that systemic vitamin D supplementation is likely to fail due to inactivation of vitamin D by locally produced CYP24A1, hampering vitamin D to reach the epithelial compartment.

Up to now, there has been only one study examining the effect of local administration of vitamin D directly into the lungs in an acute lung injury hamster model induced by LPS inhalation [39]. In this model, the effects of oral and local administration of 1α,25(OH)_2_D_3_ on neutrophil and monocyte number in BAL fluid were compared [39]. Interestingly, both oral and intra-tracheal administration of 1α,25(OH)_2_D_3_ reduced neutrophil recruitment in BAL fluid in a dose-dependent manner. However, 91% less 1α,25(OH)_2_D_3_ was used locally to reach the same 40% reduction in BAL neutrophils than with oral administration [39]. Our data showing a 51% reduction of BAL neutrophils in LPS-induced acute lung inflammation in VDS mice when treated locally with 1α,25(OH)_2_D_3_ are in line with the data of Takano et al. [39]. Moreover, in our study the local administration of 1α,25(OH)_2_D_3_ reduced BAL neutrophil by only 26% in VDD mice. However, it is important to note that the level of neutrophil infiltration in the lungs after LPS administration was higher in VDD mice compared to VDS mice. It is, therefore, possible that a higher concentration of 1α,25(OH)_2_D_3_ would be needed in VDD mice to induce a similar decrease in BAL neutrophils as in VDS mice. Increasing the local dose of 1α,25(OH)_2_D_3_ is likely feasible knowing that Takano et al. demonstrated a safe use of 1α,25(OH)_2_D_3_ orally from 1 to 10 µg/kg before showing any hypercalcemia [39], implying that such dosage would also be suitable for local administration. In summary, our data indicated that local 1α,25(OH)_2_D_3_ nebulization reduced LPS-induced acute inflammation in VDS mice without inducing hypercalcemia and higher 1α,25(OH)_2_D_3_ concentrations would be warranted in VDD mice to obtain a better anti-inflammatory effect as LPS-induced lung inflammation was more severe.

Surprisingly, while the data of CXCL5 followed the pattern of neutrophilic inflammation in VDS mice, discrepancies were present in the VDD mice. Whereas neutrophilic inflammation in BAL tended to be higher in vehicle-treated VDD compared to VDS mice, this was not associated with a higher production of CXCL5 in BAL. Similarly, the slight reduction of neutrophilic inflammation with vitamin D nebulization in VDD mice was not associated with reduction of CXCL5 with vitamin D nebulization. However, while neutrophils are produced by immune cells, CXCL5 is produced by epithelial cells which are damaged in the vehicle-treated VDD mice as shown by the severe increase in uric acid. This will impair any increase in CXCL5 in the vehicle-treated VDD mice and explain why CXCL5 did not follow the pattern of neutrophilic inflammation in VDD mice.

In this study, local 1α,25(OH)_2_D_3_ nebulization prevented to some extent the production of chemokines like CXCL5 in BAL of VDS mice. Intriguingly, IL-13 was elevated in BAL and lung of vehicle-treated VDD mice compared to vehicle-treated VDS mice and local 1α,25(OH)_2_D_3_ nebulization prevented this IL-13 production. This IL-13 production solely in the vehicle-treated VDD mice might be the consequence of low levels of circulating 1α,25(OH)_2_D_3_ with vitamin D deficiency, hampering vitamin D to inhibit LPS-induced IL-13 as previously reported [32]. On the other hand, local 1α,25(OH)_2_D_3_ nebulization in these VDD mice was likely sufficient to restore the ability of vitamin D to suppress LPS-induced IL-13 and to prevent IL-13 production in the VDD. It is, however, surprising that the higher IL-13 production in vehicle-treated VDD mice was not associated with concomitant suppression of inflammatory cytokines and chemokines as expected from study in sepsis induced by cecal ligation and puncture in VDS mice [29]. It might be speculated that cytokine and chemokine production inhibition due to IL-13 was likely disturbed by vitamin D deficiency or these data might simply reflect that endogenous IL-13 is not implicated as a modulator of local inflammation in the acute LPS lung injury model. Altogether, our data showed a slight preventive effect of local 1α,25(OH)_2_D_3_ nebulization on the production of LPS-induced pro-inflammatory cytokines and chemokines in lung and BAL.

In the current study, local 1α,25(OH)_2_D_3_ nebulization did not alter the mRNA expression of claudin-3, -5, -8 and ZO-1 in VDS mice. For the latter, this contrasts with previous data in an acute lung injury model that reported prevention of LPS-induced reduction in ZO-1 levels after systemic pre-treatment with a vitamin D analog (paricalcitolin) [40]. For claudins, there are no data available in the LPS lung injury model but in intestine, the downregulation of claudin-5 and ZO-1 due to intraperitoneal administration of LPS to yellow catfish was prevented by pre-treatment with systemic vitamin D [41]. In the current study, the absence of effect with 1α,25(OH)_2_D_3_ nebulization pre-treatment on claudin-3, -5, -8 and ZO-1 expression in VDS animals suggests that local 1α,25(OH)_2_D_3_ pre-treatment in the lung, at least at this dose, is not sufficient in preventing the LPS-induced downregulation of these claudins or ZO-1.

The most intriguing finding in our study is the reduced claudin-18 mRNA expression with 1α,25(OH)_2_D_3_ nebulization in the VDS mice. How and why pre-treatment with local 1α,25(OH)_2_D_3_ nebulization in the context of acute LPS lung injury model is impairing claudin-18 expression is unclear, with few studies exploring this topic. It is known that Claudin-18 expression is reduced in experimental LPS lung injury model [42] meaning that 1α,25(OH)_2_D_3_ nebulization in the current study worsens LPS effect on claudin-18 in VDS mice and should be associated with epithelial barrier dysfunction. In fact, data from claudin-18 deficient mice showed increased fluid clearance through increased activation of sodium and chloride channels to compensate for increased permeability, a strategy that protected the mice against lung edema [43]. Thus, in the current study, the reduced claudin-18 expression with 1α,25(OH)_2_D_3_ nebulization might eventually be regarded as a protective effect of vitamin D against LPS-induced lung injury.

To the best of our knowledge, we are the first reporting epithelial barrier permeability weakness in the vehicle- and LPS-treated VDD mice as shown by the significant reduction of ZO-1 in the lungs and severe alveolar protein leakage. This corroborates previous data in VDR knockout mice [25, 40]. The increase in uric acid in the BAL fluid in these mice indicated that epithelial damage was severe and, importantly, local 1α,25(OH)_2_D_3_ nebulization in VDD mice was able to reduce the alveolar protein leakage and epithelial damage towards VDS levels but it did not prevent the reduction of ZO-1 in the lungs. These data indicate that although inflammation was not reduced in VDD mice with local 1α,25(OH)_2_D_3_ administration, this strategy did prevent severe damage to the lungs.

This is the first time that the potential use of local 1α,25(OH)_2_D_3_ into the lungs while using a nebulization approach has been explored. This study suggests that such strategy is feasible and may be a more appropriate approach than the intratracheal instillation commonly used in mice. Furthermore, the use of 1α,25(OH)_2_D_3_ as a bronchial inhaler, that is aerosolized directly into the lungs, represents a better and effective alternative than oral supplementation for several reasons: (1) it can directly interact with the airways and immune cells; (2) low dose of 1α,25(OH)_2_D_3_ is needed; (3) dose can be increased and still remain in a safe range with no hypercalcemic effect; (4) chronic administration is likely feasible with lower risk for adverse effects; (5) the majority of the dose administered is likely to reach its target. However, caution is needed in long-term usage as both dosage and frequency need to be carefully considered in order to ensure the absence of adverse effects and the maintenance of the beneficial effects. In addition, it is still unknown whether local nebulization of 1α,25(OH)_2_D_3_ would reduce inflammation against viable pathogens, but in vitro studies have shown that exogenous 1α,25(OH)_2_D_3_ is able to boost the immune defense with the production of cathelicidin that has antiviral and antibacterial properties [23, 38, 44, 45].

Finally, this study has some limitations that need to be mentioned. First, although significant changes were observed, the sample size may appear low and have contributed to some extent to the lack of significant differences in some measurements. This is the case but solely for the measure of CXCL5 in BAL when comparing vehicle-VDS with vehicle-VDD (type II error: 0.41). For the other assessments, statistical power was above 80%. Second, the data of this study have not been replicated yet although this could further validate the data. Our pilot study at least confirms the beneficial effects of local 1α,25(OH)_2_D_3_ nebulization into the lungs in preventing cellular inflammation in BAL of VDS mice (data not shown). Lastly, it is important to keep in mind that according to our data, not only the COPD patients with severe vitamin D deficiency but also those with a normal vitamin status might benefit from this approach of 1α,25(OH)_2_D_3_ nebulization into the lungs. Although the amount of COPD patients with severe vitamin D deficiency might represent a small group, they should not be neglected knowing that severe vitamin D deficiency in COPD patients is associated with frequent exacerbations [13, 14] and exacerbations have serious repercussions on health status, lung function and quality of life.

## Conclusion

This study showed that local delivery of 1α,25(OH)_2_D_3_ directly to the lungs via nebulization was an efficient strategy to reduce acute inflammation caused by LPS both under normal vitamin D status but also in case of severe vitamin D deficiency. This approach was particularly beneficial in preventing epithelial barrier leakage and damage under severe vitamin D deficiency. More research is needed to determine the potential long-term beneficial effects of local 1α,25(OH)_2_D_3_ nebulization on lung inflammation.

## Supplementary Information

**Additional file 1.** Representative examples of sagittal lung sections stained with Hematoxylin and Eosin in LPS-treated vitamin D sufficient and deficient mice either pretreated with vehicle.

**Additional file 2.** Representative examples of sagittal lung sections stained with Hematoxylin and Eosin in LPS-treated vitamin D sufficient and deficient mice either pretreated with 1α,25(OH)2D3 nebulization.

**Additional file 3.** Primer sequences. Ribosomal protein L27 (RPL27), C-X-C motif ligand (CXCL), Claudin (Cldn), Zona occludens-1 (ZO-1).

**Additional file 4.** Overview of the assay detection range for each inflammatory mediator. Concentrations are expressed in pg/ml.

## Data Availability

All data generated or analyzed during this study are included in this published article. The datasets related to the study either used or analyzed are available from the corresponding author on reasonable request.

## Abbreviations

AECOPD: Acute exacerbation of chronic obstructive pulmonary disease
ARTI: Acute respiratory tract infection
BAL: Broncho-alveolar lavage
cDNA: Complementary deoxyribonucleic acid
Cldn: Claudin
COPD: Chronic Obstructive Pulmonary Disease
CXCL: Chemokine (C-X-C motif) ligand
CYP24A1: Cytochrome P450 Family 24 Subfamily A Member 1
CYP27B1: Cytochrome P450 Family 27 Subfamily B Member 1
FEV_1_: Forced Expiratory Volume in 1s
IFN-ϒ: Interferon gamma
IL: Interleukin
IQR: Interquartile range
LABA: Long acting beta2 agonist
LAMA: Long acting antimuscarinic antagonist
LPS: Lipopolysaccharide
mRNA: Messenger ribonucleic acid
NS: Not significant
PCR: Polymerase chain reaction
qRT-PCR: Quantitative real time polymerase chain reaction
RNA: Ribonucleic acid
RPL27: Ribosomal protein L27
TNF-α: Tumor necrosis factor alpha
VDD: Vitamin D deficient
VDR: Vitamin D receptor
VDS: Vitamin D sufficient
ZO-1: Zona occludens-1
1α,25 (OH)_2_D: 1α,25-dihydroxyvitamin D
1α,25 (OH)_2_D_3_: 1α,25-dihydroxyvitamin D3
25(OH)D: 25-Hydroxyvitamin D
