# Supplementary material for: Local nebulization of 1α,25(OH)2D3 attenuates LPS-induced acute lung inflammation
Source: Respir Res. 2022 Mar 29;23:76. doi: 10.1186/s12931-022-01997-9 (PMC8966160; doi:10.1186/s12931-022-01997-9)
Supplement: Supplementary file 1 — Additional file 1. Representative examples of sagittal lung sections stained with Hematoxylin and Eosin in LPS-treated vitamin D sufficient and deficient mice either pretreated with vehicle. [file 12931_2022_1997_MOESM1_ESM.docx]

Table S1: Primer sequences. Ribosomal protein L27 (RPL27), C-X-C motif ligand (CXCL), Claudin (Cldn), Zona occludens-1 (ZO-1).

| Target | Forward primer | Reverse primer | Accession ID | |
| --- | --- | --- | --- | --- |
| RPL27 | 5’- GTCGAGATGGGCAAGTTCAT-3’ | 5’-TTCTTCACGATGACGGCTTT-3’ | | NM_011289.3 |
| CXCL1 | 5’-ACCGAAGTCATAGCCACACTC-3’ | 5’-TCTCCGTTACTTGGGGACAC-3’ | | [NM_008176.3](https://www.ncbi.nlm.nih.gov/nucleotide/229577225?report=genbank&log$=nucltop&blast_rank=1&RID=SSV9FGZS015) |
| CXCL2 | 5’-TGAACTGCGCTGTCAATGC -3’ | 5’- GCTTCAGGGTCAAGGCAAAC-3’ | | NM_009140.2 |
| CXCL5 | 5’-TGGATCCAGAAGCTCCTGTGA-3’ | 5’- TGCATTCCGCTTAGCTTTCTTT-3’ | | NM_009141.3 |
| Cldn3 | 5’-AAGCCGAATGGACAAAGAA-3’ | 5’-CTGGCAAGTAGCTGCAGTG -3’ | | NM_009902.4 |
| Cldn5 | 5’-GTGGAACGCTCAGATTTCAT-3’ | 5’-TGGACATTAAGGCAGCATCT -3’ | | NM_013805.4 |
| Cldn8 | 5’-GGTTCACTCAGCTCTTCCTTT-3’ | 5’-CACCCGCATCTACTTGGATAAT -3’ | | NM_018778.3 |
| Cldn18 | 5’-TCCACGGCCTTAGTACTCATA -3’ | 5’-CCAGACACACAGGTCTCTATTG -3’ | | NM_019815.3 |
| ZO-1 | 5’- AGGTCTTCGCAGCTCCAAGAGAAA -3’ | 5’- ATCTGGCTCCTCTCTTGCCAACTT-3’ | | NM_0011633574.1 |
