# Supplementary material for: Local nebulization of 1α,25(OH)2D3 attenuates LPS-induced acute lung inflammation
Source: Respir Res. 2022 Mar 29;23:76. doi: 10.1186/s12931-022-01997-9 (PMC8966160; doi:10.1186/s12931-022-01997-9)
Supplement: Supplementary file 2 — Additional file 2. Representative examples of sagittal lung sections stained with Hematoxylin and Eosin in LPS-treated vitamin D sufficient and deficient mice either pretreated with 1α,25(OH)2D3 nebulization. [file 12931_2022_1997_MOESM2_ESM.docx]

Table S2 Overview of the assay detection range for each inflammatory mediator. Concentrations are expressed in pg/ml.

| Mediator | IFN-γ | IL-1β | IL-4 | IL-6 | IL-10 | IL-13 | IL-17A | TNF-α | CXCL1 | CXCL2 | CXCL5 |
| --- | --- | --- | --- | --- | --- | --- | --- | --- | --- | --- | --- |
| Range | 0.16-2,900 | 3.1-13,000 | 0.56-10,000 | 4.8-16,000 | 3.8-22,800 | 2.7-22,800 | 0.3-2,150 | 1.3-6,200 | 4.8-16,000 | 0.21-2,100 | 15.6-1,000 |
