# Supplementary figures and images for: Local nebulization of 1α,25(OH)2D3 attenuates LPS-induced acute lung inflammation
Source: Respir Res. 2022 Mar 29;23:76. doi: 10.1186/s12931-022-01997-9 (PMC8966160; doi:10.1186/s12931-022-01997-9)

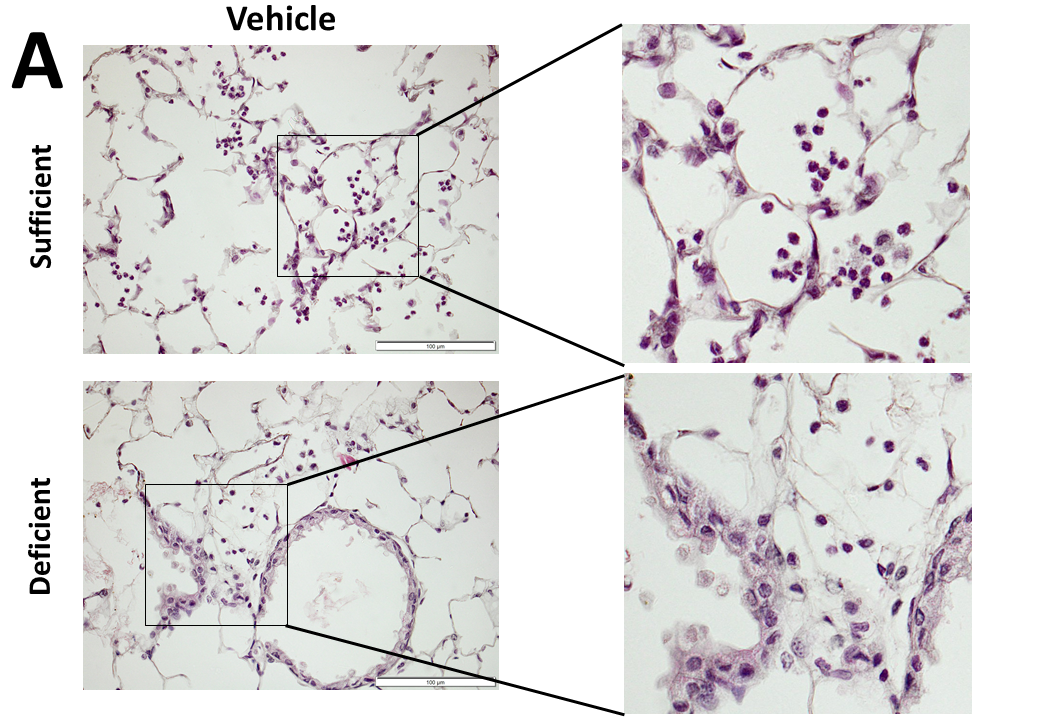

Supplement: Supplementary file 3 — Additional file 3. Primer sequences. Ribosomal protein L27 (RPL27), C-X-C motif ligand (CXCL), Claudin (Cldn), Zona occludens-1 (ZO-1). [file 12931_2022_1997_MOESM3_ESM.tif]

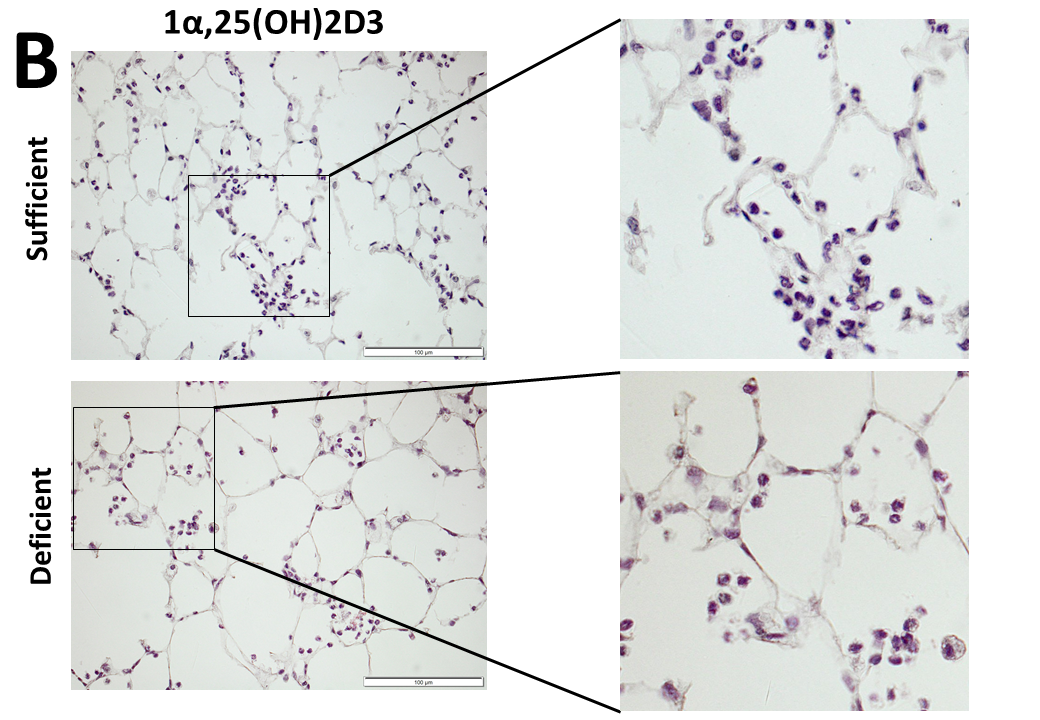

Supplement: Supplementary file 4 — Additional file 4. Overview of the assay detection range for each inflammatory mediator. Concentrations are expressed in pg/ml. [file 12931_2022_1997_MOESM4_ESM.tif]
